# A Novel Decision Tree Model for Predicting the Cancer-Specific Survival of Patients with Bladder Cancer Treated with Radical Cystectomy

**DOI:** 10.3390/jcm13082177

**Published:** 2024-04-10

**Authors:** Pau Sarrio-Sanz, Laura Martinez-Cayuelas, Abraham Beltran-Perez, Milagros Muñoz-Montoya, Jose-Vicente Segura-Heras, Vicente F. Gil-Guillen, Luis Gomez-Perez

**Affiliations:** 1 Urology Services, University Hospital of San Juan de Alicante, 03550 San Juan de Alicante, Alicante, Spain; martinezcayuelaslaura@gmail.com (L.M.-C.); milamumo95@gmail.com (M.M.-M.); 2 Public Health, Science History and Gynaecology Department, Miguel Hernández University, 03550 San Juan de Alicante, Alicante, Spain; abeltran@umh.es; 3 Instituto Centro de Investigación Operativa, Miguel Hernández University, 03550 Elche, Alicante, Spain; jvsh@umh.es; 4 Clinical Medicine Department, Miguel Hernández University, 03550 San Juan de Alicante, Alicante, Spain; vte.gil@gmail.com; 5 Pathology and Surgery Department, Miguel Hernández University, 03550 San Juan de Alicante, Alicante, Spain; luisgope@gmail.com; 6 Urology Services, University and General Hospital of Elche, 03203 Elche, Alicante, Spain

**Keywords:** bladder cancer, mortality, predictions and projections, decision trees

## Abstract

**Background/Objectives**: The aim was to develop a decision tree and a new prognostic tool to predict cancer-specific survival in patients with urothelial bladder cancer treated with radical cystectomy. **Methods**: A total of 11,834 patients with bladder cancer treated with radical cystectomy between 2004 and 2019 from the SEER database were randomly split into the derivation (*n* = 7889) and validation cohorts (*n* = 3945). Survival curves were estimated using conditional decision tree analysis. We used Multiple Imputation by Chained Equations for the treatment of missing values and the pec package to compare the predictive performance. We extracted data from our model following CHARMS and assessed the risk of bias and applicability with PROBAST. **Results**: A total of 4824 (41%) patients died during the follow-up period due to bladder cancer. A decision tree was made and 12 groups were obtained. Patients with a higher AJCC stage and older age have a worse prognosis. The risk groups were summarized into high, intermediate and low risk. The integrated Brier scores between 0 and 191 months for the bootstrap estimates of the prediction error are the lowest for our conditional survival tree (0.189). The model showed a low risk of bias and low concern about applicability. The results must be externally validated. **Conclusions**: Decision tree analysis is a useful tool with significant discrimination. With this tool, we were able to stratify patients into 12 subgroups and 3 risk groups with a low risk of bias and low concern about applicability.

## 1. Introduction

Urothelial bladder cancer (UBC) is the second most common urological malignancy, with a 2–7.1 (per 100,000) annual age-standardized mortality rate in the United States and 10.1 in Europe [1,2,3]. Despite mortality rates decreasing, there are expected to be almost 16,700 deaths due to bladder cancer in the United States at 2023 [4,5]. At 5 years, the cancer-specific survival for patients with muscle invasive ranges from 23.5% to 65%, depending on the study [6].

Radical cystectomy (RC) with bilateral pelvic lymph node dissection preceded by neoadjuvant cisplatin-based chemotherapy is the standard of care treatment for non-metastatic muscle invasive UBC. Patients with a high or very high risk of non-muscle invasive UBC are also candidates for RC, usually without neoadjuvant cisplatin-based chemotherapy [7,8].

RC is associated with high morbidity (25% to 35%) and mortality rates (0.7% to 11%) [9].

There is a strong association between the pathological TNM stage and cancer-specific survival (CSS); however, is not enough to predict the prognosis for most patients. There are other important items to consider, such as age, sex, race, surgical margins or tumor size [10,11,12]. In addition, preoperative treatments as neoadjuvant chemotherapy can safely improve survival outcomes in comparison to the use of locoregional treatment alone [13]. When a patient presents a prognostic factor for a disease, the probability of death increases. To estimate this probability, we need mathematical prognostic models to determine the risk of death for each individual patient.

Due to the significant recurrence rate after RC, several predictive models have been developed in patients with UBC treated with RC to predict CSS. All these models have been systematically reviewed and summarized previously [6]. In this systematic review, the authors provide a synthesis of the 19 prediction models identified. Among them, 52.6% showed low applicability and all of them had a high risk of bias; these findings agree with previous systematic reviews carried out with a similar methodology [6,14]. 

Therefore, there remains a need to construct and validate a new prognostic tool to identify patients with a high risk of dying due to UBC, since these patients might be candidates for intensive postoperative surveillance, adjuvant therapy or potential clinical trials [15]. The current study aims to develop and internally validate a decision tree and a new prognostic tool to predict CSS in patients with UBC treated with RC. 

## 2. Materials and Methods

### 2.1. Patients

The primary data of patients with UBC treated with RC were obtained from the Surveillance, Epidemiology and End Results (SEER) database utilizing the 2000 to 2019 SEER research data. SEER contains data collected by 17 population-based registries, which cover approximately 26.5% of the US population. Institutional review board approval was obtained from our institution (AUT.DPC.LGP.01.22). 

The inclusion criteria for this study were as follows: diagnosed as urinary bladder cancer (C670–679) between 2004 and 2019, urothelial carcinoma histology (International Classification of Diseases for Oncology: 8120) and treated with radical cystectomy (codes 60, 61, 62, 63, 64, 71). 

The base was requested for the following variables: age, sex, race, year of diagnosis, summary stage, AJCC (6, 7, 8 version), TNM stage, SEER cause-specific death classification, tumor size (millimeters), tumor grade (low and high grade), survival time (months) and vital status. The AJCC stage was reclassified according to the last version of the AJCC classification. 

The summary stage variable summarizes the real state of the patients in the groups: in situ, localized, regional by direct extension and/or regional lymph nodes involved and distant sites or lymph nodes involved [16]. The T stage was simplified using the subheadings T1, 2, 3 and 4. The N and M stages were simplified as positive or negative. We excluded data about radiotherapy or chemotherapy because in the SEER database, chemotherapy data are categorized as either “yes—patient had chemotherapy” or “no/unknown” and there is a lack of data regarding when was the patient treated (neo or adjuvant treatments).

Patients with less than 3 years of follow-up were excluded. The primary endpoint was CSS. The survival time was calculated from the date of RC to the date of death from UBC.

### 2.2. Statistical Analysis

The descriptive statistical analysis included the mean and standard deviation for the continuous variables and the counts and percentages for the categorical variables. We have considered conditional inference trees for survival analysis with censored data, which do not assume the need for proportional hazards and have the flexibility to model curves with different shapes for identified groups of subjects. Such trees estimate a regression relationship by recursive binary partitioning in a conditional inference structure, which ensures adequate tree growth without the need for further cross-validation [17]. The algorithm works in three steps. (1) It tests the value of the global hypothesis of independence between the input variables and the answer (which can also be multivariate), stopping the algorithm if it cannot reject the hypothesis. Otherwise, selecting the input variable with the strongest association with the answer. The *p*-value measures this association corresponding to a test for the partial null hypothesis of a single input variable and the answer. (2) It implements a binary division on the selected input variable. (3) It repeats steps 1 and 2 several times [17].

To perform the predictive model, we selected 2/3 of the sample (derivation cohort), and we confirmed the model’s validity by applying the parameters to 1/3 of the remaining sample (validation cohort).

There are several works where we can see the advantages of using this type of tree compared to the classical Cox regression models for proportional hazards [18,19].

We used the *p*-value adjusted log-rank statistics proposed by Schumacher et al. [20] for the evaluation of the prognostic factors. We have generated multiple imputations for incomplete data using chained equations (MICE) using a classification tree if the variable is qualitative and a regression tree when the variable is quantitative. We have used the pec package to compare the predictive performance of our proposal with the covariate-free survival Kaplan–Meier model and the Cox regression model, through the error defined as the time-dependent expected Brier score [21]. We considered for its calculation 500 samples of size 7889 that were randomly obtained from our database with 11,834 records using a bootstrap cross-validation process. The statistical analysis was performed with the statistical package R, version 3.6.3. A two-tailed *p* < 0.05 was considered statistically significant.

Finally, a blinded author assessed the risk of bias and applicability with the Prediction model study Risk Of Bias Assessment Tool (PROBAST) [22] and extracted relevant items from this study following the Critical Appraisal and Data Extraction for Systematic Reviews of Prediction Modelling (CHARMS) to identify potential sources of bias and summarize the model characteristics [23].

## 3. Results

Finally, 11,834 patients with UBC treated with RC were included. Of them, 77% (*n* = 9072) were male. The mean age was 68 years. A total of 4824 (41%) patients died during the follow-up period due to UBC. The mean survival time was 97 months (CI 95%: 87–123).

Table 1 shows the characteristics of the patients in each of the samples considered, training and validation. It can be seen that the randomization was performed correctly because no significant differences were detected between them (*p*-value > 0.05). In addition, the last column shows the percentage of missing values for each variable. The variables with the highest percentage of missing values are size (27.09%) and tumor grade (11.35%), followed by AJCC stage (8%) and stage N (1.40%).

**Figure 1:**
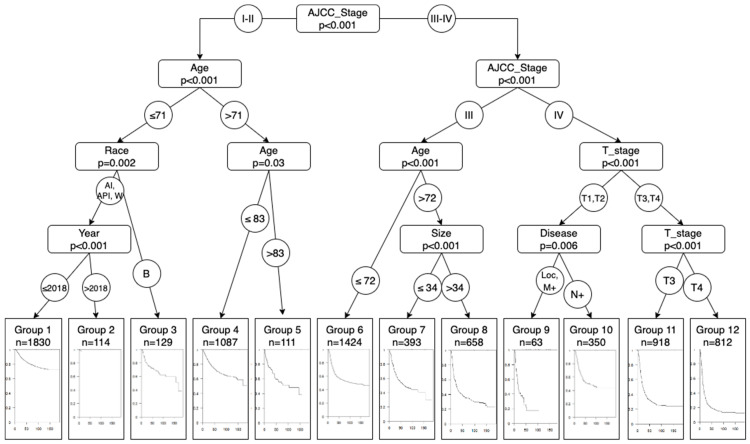
Conditional inference tree with derivation cohort. Following this figure, we can classify and stratify the risk of death due to bladder cancer of our patients. The final image is the survival curve using the conditional inference tree for each subgroup of patients and the number of patients included in each subgroup.

**Figure 2:**
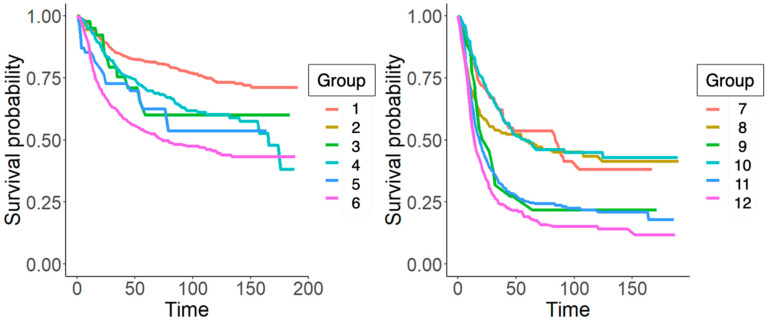
Conditional inference tree with validation cohort. In this figure, the authors present the survival curves for each subgroup of patients included in the study cohort.

We obtained 12 risk groups with significant differences in their mortality (*p* < 0.01). As we can see from Figure 1, to split the patients into different risk groups, the most important variable is the AJCC stage. The survival curves (Kaplan–Meier curves) of each subgroup created in Figure 1 have been drawn in Figure 2 to simplify the comparison between subgroups.

The patients with the best prognosis are those under 71 years of age, with AJCC stages I or II, who are not Black. Black patients have a worse prognosis (group 3), comparable to that of patients above the age of 83 (group 5). The prognosis is slightly better when these patients are between 71 and 83 years of age.

The worst prognoses involve patients with stages IV and T3 (group 11) or T4 (group 12). Those with stages T1 or T2 and positive lymph nodes have a better prognosis (group 10) than those with localized or metastatic disease (group 9).

For the group of patients with AJCC stage III, we can distinguish between patients below the age of 72 (group 6) and those above the age of 72 with a tumor smaller than 34 mm (group 7).

Table 2 shows the number of cases identified for each group, the events that have occurred, the estimated mean survival times, considering the upper limit of survival, as well as the median survival and its 95% confidence interval, in those cases where it is possible to estimate it. Groups 4 and 11 have the lowest number of cases.

A possible performance measure for the classification tree can be obtained by discretizing the response variable into the high, intermediate, and low risk of surviving the disease, considering the 40th and 50th percentiles of the survival model. Using this criterion, groups 1 to 6 would be classified as low risk, groups 7 and 10 as intermediate risk, while groups 8, 9, 11 and 12 would be associated with a high risk.

**Figure 3:**
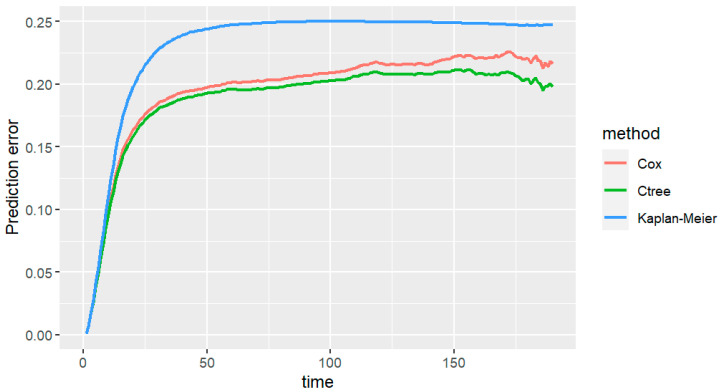
Variation in the prediction error over time for each alternative. In this image, we compare the area under the curve using different statistical approaches for the acquisition of the survival curves (conditional inference, Cox regression and Kaplan–Meier curves). The prediction error is lower for our conditional survival tree (0.189) than if we performed it with the Cox regression model (0.197) or Kaplan–Meier model (0.231).

The integrated Brier scores between 0 and 191 months for the bootstrap estimates of the prediction error are lower for our conditional survival tree (0.189) than if we performed it with the Cox regression model (0.197) or Kaplan–Meier model (0.231) (Figure 3).

Data about the model were extracted following the CHARMS items (Table 3). According to PROBAST tool, the model has a low risk of bias and low concern about applicability (Supplemental Material S1).

## 4. Discussion

The decision tree presented in this paper has been developed and validated internally in a national cohort of more than 11,800 patients to predict CSS in patients undergoing RC. Finally, 12 risk groups have been created, which can be further categorized into 3 major groups. The model provides meaningful discrimination and is easily applicable in clinical practice.

Bladder cancer results in patient death at a median of 97 months after RC. During the follow-up period, two in five patients died due to urothelial bladder cancer. However, the outcome of bladder cancer is heterogeneous depending on the clinical characteristics of each patient, so clinicians need validated tools to help estimate cancer-specific survival to enable them, for example, to use adjuvant multimodal therapies, inform patients and their families of disease severity, individualized follow-up schedules, or stratify patients in clinical trials.

Previously, multiple predictive models had been developed to stratify or personalize the risk of each patient [6], but all of them had a high risk of bias and uncertain applicability. Actually, there is no consensus on the ideal follow-up schedule for these patients and the early detection of recurrences. Therefore, stratifying patients according to risk is the first step in defining the appropriate follow-up regimen.

This model is the first to use decision trees in genitourinary tumors. This design provides several advantages over classical Cox regression models, such as the higher prediction accuracy with consequent statistical robustness and transparency [18]. It has been used to obtain simpler and more intuitive models in other pathologies, such as breast cancer [17], thus facilitating clinical application and providing less prediction error over time.

The division into three groups was carried out heuristically. Previous studies divided risk into slight or low risk based on the comparison of each group with the overall median. In this case, the authors have considered introducing an intermediate risk option, which fits the interval associated with the 40th and 50th percentiles. This results in a high-risk group with a survival of less than 2 years (24 months), an intermediate risk group with a mean survival of around 5 years (60 months), and finally, a low-risk group with a mean survival of more than 100 months (more than 8 years).

The prognostic factors related to cancer-specific survival are the AJCC stage, age, T stage, tumor size, race, and year of diagnosis. Previous studies have shown that many factors can influence CSS after cystectomy [6]. The variable “AJCC Stage” has previously been used in multiple studies in bladder cancer with patients from the SEER database [10,12]. It is a very easy variable to apply in clinical practice and, as seen in this study, it has the highest discriminatory power.

In our model, age is an independent risk factor for mortality, such that older patients have a worse prognosis. Most previously published models agree with our data, with younger patients having a better prognosis [8,12,24,25,26]; however, Di Trapani et al. [27] developed a model in 2015 indicating that younger patients had a higher risk of dying from bladder cancer in the first 3 years after surgery than older patients.

The local T stage is an important prognostic factor. As in our work, it has been included in the vast majority of models designed to date, except those designed by Gondo et al. and May et al. [28,29]. The higher the T stage at the time of cystectomy, the higher the risk in most published models. Di Trapani et al. reported a worse prognosis in T2 patients compared to ≥T3, probably because they used the stage measured after bladder trans urethral resection instead of the stage measured at the time of cystectomy [27].

A larger tumor size is an indicator of worse prognoses in the patients studied, a fact already observed by Yang et al. and Gondo et al. [10,28]. In this model, the cut-off point of 34 mm was given following mathematical models. This is a subjective cut-off point and is between the cut-off point proposed by Yang et al. (40 mm) and Gondo et al. (30 mm) [10,28]. We used linear analysis instead of categorizations, which reduces the risk of bias following the CHARMS and PROBAST recommendations.

Race has not been included as a candidate variable in most studies designed to date; however, a worse prognosis in Black patients has been reported previously [12], probably due to socioeconomic differences and access to healthcare in the United States.

Unlike other previously published models [12,24,30], and following the results of Mir et al. [11], sex is not an independent risk factor in the present study.

During the last few years, emerging novel treatments, including antibody–drug conjugates and immunotherapy targeted treatments, have been approved for cisplatin-ineligible patients or after recurrence [15]. As we can see in our results, patients diagnosed before 2018 will have worst survival than patients nowadays. These results agree with previous studies, with the year of diagnosis included as a main predictor of cancer-specific survival [10].

The main strength of this model is the large number of patients included and the use of clinical and pathological variables previously suggested by other studies, which are objective and easily measurable in routine clinical practice. However, as a limitation, other previously described factors, such as the lymphovascular invasion, hemoglobin values, positive surgical margins or the number of nodes removed, have not been included in the study due to the absence of these parameters in the registry used.

This study was conducted on a representative cohort of the United States population. However, our study suffers from some limitations inherent to the retrospective design, especially in the completion of the following variables. A selection bias may exist because this database does not collect patients from all American hospitals and also due to the loss of follow-up or missing data for some patients (especially for the size and grade variables). To reduce the risk of bias due to missing data, a series of mathematical calculations were performed to impute missing data.

On the other hand, unifying the different TNM classifications could lead to a classification bias. In the SEER database, the classification is based on the sixth, seventh and eighth editions of the AJCC. Over the years, the T and M classifications have not changed substantially. As regards the N classification, there have been changes in its subclassifications (N1, N2, etc.). So, to reduce the possibility of bias, the decision was taken to unify patients with positive nodes into a single subgroup.

This model has been validated internally. However, the model was derived from patients belonging to a specific healthcare system, so future research is needed to validate the decision tree in other countries using the PROBAST methodology [22]. After external validation in each geographical area, this model could be useful for counselling and informing patients, as well as making decisions about the adjuvant treatment and risk stratification of patients, especially in clinical trials.

This study shows that the risk of prediction error is lower with the decision tree methodology. Therefore, predictive models should be developed using this methodology for use in the follow-up of other patients with bladder cancer and other genitourinary tumors.

The current model can be used for patients undergoing RC in the United States. The subclassification of patients into 3 risk groups (with 12 subgroups) could be useful for stratifying patients participating in clinical trials.

This model does not include patients with histological variants other than urothelial cancer, so its use in this group of patients would not be recommended.

## 5. Conclusions

In conclusion, patients with AJCC stages III and IV and older patients have worse prognoses. Decision trees are a very useful tool and provide significant discrimination, allowing patients to be grouped into 3 broad risk groups and 12 other subgroups. After applying PROBAST as the gold standard to verify that the model was correctly developed, our decision tree has a positive applicability score and a low risk of bias. These results need to be externally validated.

## Figures and Tables

**Table 1:** Patient characteristics and missing values of the patients included in the study.

Variable	Derivation Cohort	Validation Cohort	Overall	*p*-Value	Missing Values (%)
	*N* = 7889	*N* = 3945	*N* = 11,834		
Sex				0.1	0
Female	1808 (23%)	954 (24%)	2762 (23%)		
Male	6081 (77%)	2991 (76%)	9072 (77%)		
Year of diagnostic				0.8	0
2004	445 (5.6%)	244 (6.2%)	689 (5.8%)		
2005	446 (5.7%)	229 (5.8%)	675 (5.7%)		
2006	486 (6.2%)	246 (6.2%)	732 (6.2%)		
2007	536 (6.8%)	251 (6.4%)	787 (6.7%)		
2008	524 (6.6%)	270 (6.8%)	794 (6.7%)		
2009	517 (6.6%)	233 (5.9%)	750 (6.3%)		
2010	565 (7.2%)	305 (7.7%)	870 (7.4%)		
2011	505 (6.4%)	268 (6.8%)	773 (6.5%)		
2012	458 (5.8%)	225 (5.7%)	683 (5.8%)		
2013	474 (6.0%)	255 (6.5%)	729 (6.2%)		
2014	527 (6.7%)	251 (6.4%)	778 (6.6%)		
2015	491 (6.2%)	238 (6.0%)	729 (6.2%)		
2016	519 (6.6%)	239 (6.1%)	758 (6.4%)		
2017	497 (6.3%)	237 (6.0%)	734 (6.2%)		
2018	461 (5.8%)	250 (6.3%)	711 (6.0%)		
2019	438 (5.6%)	204 (5.2%)	642 (5.4%)		
Age	68.06 (9.98)	68.11 (10.05)	68.08 (10.00)	0.7	0
Race				0.1	0.19
American Indian/Alaska Native	33 (0.4%)	24 (0.6%)	57 (0.5%)		
Asian or Pacific Islander	353 (4.5%)	206 (5.2%)	559 (4.7%)		
Black	486 (6.2%)	220 (5.6%)	706 (6.0%)		
White	7017 (89%)	3495 (89%)	10,512 (89%)		
Death classification				0.7	0.7
Alive or death of another cause	4664 (59%)	2346 (59%)	7010 (59%)		
Death due to bladder cancer	3225 (41%)	1599 (41%)	4824 (41%)		
Survival time (month)	47.38 (46.21)	47.64 (47.10)	47.47 (46.51)	0.8	0
Summary stage				0.1	0.62
Localized	3204 (41%)	1603 (41%)	4807 (41%)		
Lymph node	4066 (52%)	2074 (53%)	6140 (52%)		
Metastatic	619 (7.9%)	268 (6.8%)	887 (7.5%)		
T stage				0.2	0.87
T1	712 (9%)	338 (8.6%)	1050 (8.9%)		
T2	3032 (38%)	1539 (39%)	4571 (39%)		
T3	2639 (33%)	1370 (35%)	4009 (34%)		
T4	1506 (19%)	698 (18%)	2204 (19%)		
N stage				0.6	1.4
Negative	5785 (73%)	2874 (73%)	8659 (73%)		
Positive	2104 (27%)	1071 (27%)	3175 (27%)		
M stage				0.9	0.59
Negative	7587 (96%)	3793 (96%)	11,380 (96%)		
Positive	302 (3.8%)	152 (3.9%)	454 (3.8%)		
Size	41.85 (36.73)	41.78 (36.95)	41.83 (36.80)	0.9	27.09
Grade				0.5	11.35
I	26 (0.3%)	15 (0.4%)	41 (0.3%)		
II	133 (1.7%)	75 (1.9%)	208 (1.8%)		
III	2014 (26%)	1047 (27%)	3061 (26%)		
IV	5716 (72%)	2808 (71%)	8524 (72%)		
AJCC stage				0.8	0
I	670 (8.5%)	316 (8%)	986 (8.3%)		
II	2601 (33%)	1319 (33%)	3920 (33%)		
III	2475 (31%)	1239 (31%)	3714 (31%)		
IV	2143 (27%)	1071 (27%)	3214 (27%)		

**Table 2:** Principal parameters for each subgroup, number of patients (*n*), number of events (events), * restricted mean with upper limit, median survival and confidence interval. Derivation (validation).

Group	*n*	Events	* r Mean	Median Survival	0.95 CI
1	1830 (885)	369 (175)	151.0 (151.4)		
2	114 (56)	2 (2)	10.8 (10.7)		
3	129 (46)	40 (11)	121.1 (124.2)	169	111–(
4	1087 (583)	288 (161)	128.5 (124.9)	(166)	171–(157-)
5	111 (65)	42 (20)	91.2 (103.4)	104	47–(57-)
6	1424 (741)	548 (312)	106.5 (100.8)	112 (74)	77–(58–122)
7	393 (183)	153 (68)	92.2 (87.3)	67 (84)	47–126 (41-)
8	658 (315)	357 (138)	68.5 (93.3)	26 (59)	22–32 (28-)
9	63 (29)	40 (18)	34.0 (54.9)	17 (27)	14–41 (16–64)
10	350 (180)	169 (87)	100.15 (99.1)	68 (58)	42–(41-)
11	918 (478)	596 (335)	63.5 (55.1)	22 (19)	20–25 (16–22)
12	812 (384)	621 (272)	41.1 (42)	14 (14)	13–15 (13–17)

**Table 3:** Relevant items extracted from the included decision tree model for predicting the cancer-specific survival of patients with bladder cancer treated with radical cystectomy based on the Critical Appraisal and Data Extraction for Systematic Reviews of Prediction Modelling Studies checklist.

CHARMS Items	Sarrio et al., 2024
Source of data	Retrospective cohort study
Participants	SEER database The total records were randomly split into development and validation cohorts in a ratio of 2:1 Patients with urothelial BC who received RC + LND Treatment is not included as a candidate predictor Baseline data: 2004–2019
Outcome to be predicted	3-year CCS Measurement: medical records Blinding unknown
Candidate predictors	Predictors: age, sex, race, year of diagnosis, tumor size, grade, AJCC stage, TNM stage and summary stage Measurement: clinical records at baseline (diagnosis) Blinding of measurement unknown, but all of them are objective Continuous predictors: linear
Sample size	*N* = 7889 (E = 3225) to develop the model and 3945 to validate it (E = 1599) EPV = 4824/35 = 168
Missing data	Multiple Imputation by Chained Equations
Model development	Conditional inference trees for survival analysis Assumptions were not tested Method for selection of predictors for inclusion in multivariable modeling: full model Method for selection of predictors during multivariable modeling: full model No shrinkage or penalization
Model performance	Discrimination: none Calibration: none Classification measures: pec package
Model evaluation	Internal: Brier score External: Brier score
Results	Indicated: model coefficients, Brier score Not indicated: none Presentation: decision tree + risk score The authors did compare the distribution of the predictors for development and validation data sets
Interpretation and discussion	Exploratory results Comparison with previous models and explanation for the predictors of the final models They analyzed strengths and limitations They discussed generalizability in other areas

Abbreviations: CHARMS, Critical Appraisal and Data Extraction for Systematic Reviews of Prediction Modelling Studies; BC, bladder cancer; RC, radical cystectomy; LND, lymph node disease; AJCC, American Joint Committee on Cancer; E, total number of events; EPV, events per variable ratio; *N*, total number of patients. The EPV was calculated using the predictors selected during multivariable modeling.

## Data Availability

No new data were created or analyzed in this study. Data sharing is not applicable to this article.

## Supplementary Materials

The following supporting information can be downloaded at https://www.mdpi.com/article/10.3390/jcm13082177/s1. Supplementary Material S1: Checklist for the evaluation of the model developed according to Prediction model study Risk Of Bias Assessment Tool.
