# Supplementary material for: A Novel Decision Tree Model for Predicting the Cancer-Specific Survival of Patients with Bladder Cancer Treated with Radical Cystectomy
Source: J Clin Med. 2024 Apr 10;13(8):2177. doi: 10.3390/jcm13082177 (PMC11050271; doi:10.3390/jcm13082177)
Supplement: Supplementary file 1 [file jcm-13-02177-s001.zip › jcm-2931142-supplementary.pdf]

Supplemental Material S1: Checklist for the evaluation of the model developed according to Prediction model study Risk Of Bias Assessment Tool.

PROBAST

(Prediction model study Risk Of Bias Assessment Tool)

Published in Annals of Internal Medicine (freely available):

1. PROBAST: A Tool to Assess the Risk of Bias and Applicability of Prediction Model Studies
2. PROBAST: A Tool to Assess Risk of Bias and Applicability of Prediction Model Studies: Explanation and Elaboration

What does PROBAST assess?

PROBAST assesses both the *risk of bias* and *concerns regarding applicability* of a study that evaluates (develops, validates or updates) a multivariable diagnostic or prognostic prediction model. It is designed to assess primary studies included in a systematic review.

Bias occurs if systematic flaws or limitations in the design, conduct or analysis of a primary study distort the results. For the purpose of prediction modelling studies, we have defined *risk of bias* to occur when shortcomings in the study design, conduct or analysis lead to systematically distorted estimates of a model's predictive performance or to an inadequate model to address the research question. Model predictive performance is typically evaluated using calibration, discrimination and sometimes classification measures, and these are likely inaccurately estimated in studies with high risk of bias. *Applicability* refers to the extent to which the prediction model from the primary study matches your systematic review question, for example in terms of the participants, predictors or outcome of interest.

A primary study may include the development and/or validation or update of more than one prediction model. A PROBAST assessment should be completed for each distinct model that is developed, validated or updated (extended) for making individualised predictions. Where a publication assesses multiple prediction models, only complete a PROBAST assessment for those models that meet the inclusion criteria for your systematic review. Please note that subsequent use of the term "model" includes derivatives of models, such as simplified risk scores, nomograms, or recalibrations of models.

PROBAST is not designed for all multivariable diagnostic or prognostic studies. For example, studies using multivariable models to identify predictors associated with an outcome but not attempting to develop a model for making individualised predictions are not covered by PROBAST.

PROBAST includes four steps.

Step	Task	When to complete
**1**	Specify your systematic review question(s)	Once per systematic review
**2**	Classify the type of prediction model evaluation	Once for each model of interest in each publication being assessed, for each relevant outcome
**3**	Assess risk of bias and applicability	Once for each development and validation of each distinct prediction model in a publication
**4**	Overall judgment	Once for each development and validation of each distinct prediction model in a publication

If this is your first time using PROBAST, we strongly recommend reading the detailed explanation and elaboration (E&E, see link above) paper and to check the examples on www.probast.org

Step 1: Specify your systematic review question

State your systematic review question to facilitate the assessment of the applicability of the evaluated models to your question. *The following table should be completed once per systematic review.*

Criteria	Specify your systematic review question
Intended use of model:	*To predict cancer specific mortality in patients with bladder cancer treated with radical cystectomy*
***Participants** including selection criteria and setting:*	*Patients with bladder cancer treated with radical cystectomy*
***Predictors** (used in prediction modelling), including types of predictors (e.g. history, clinical examination, biochemical markers, imaging tests), time of measurement, specific measurement issues (e.g., any requirements/ prohibitions for specialized equipment):*	*Predictors used in clinical practice measured when a cystectomy for bladder cancer is indicated*
Outcome to be predicted:	*Cancer specific mortality*

Step 2: Classify the type of prediction model evaluation

Use the following table to classify the evaluation as model development, model validation or model update, or combination. Different signalling questions apply for different types of prediction model evaluation. If the evaluation does not fit one of these classifications then PROBAST should not be used.

**Classify the evaluation based on its aim**			
**Type of prediction study**	**PROBAST boxes to complete**	**Tick as appropriate**	**Definition for type of prediction model study**
Development only	Development	✗	Prediction model development without external validation. These studies may include internal validation methods, such as bootstrapping and cross-validation techniques.
Development and validation	Development and validation	✓	Prediction model development combined with external validation in other participants in the same article.
Validation only	Validation	✗	External validation of existing (previously developed) model in other participants.

This table should be completed once for each publication being assessed and for each relevant outcome in your review.

**Publication reference**	Sarrio et al.,
**Models of interest**	Risk score, conditional inference tree
**Outcome of interest**	*Cancer specific survival*

Step 3: Assess risk of bias and applicability

PROBAST is structured as four key domains. Each domain is judged for risk of bias (low, high or unclear) and includes signalling questions to help make judgements. Signalling questions are rated as yes (Y), probably yes (PY), probably no (PN), no (N) or no information (NI). All signalling questions are phrased so that "yes" indicates absence of bias. Any signalling question rated as "no" or "probably no" flags the potential for bias; you will need to use your judgement to determine whether the domain should be rated as "high", "low" or "unclear" risk of bias. The guidance document contains further instructions and examples on rating signalling questions and risk of bias for each domain.

The first three domains are also rated for concerns regarding applicability (low/ high/ unclear) to your review question defined above.

Complete all domains separately for each evaluation of a distinct model. Shaded boxes indicate where signalling questions do not apply and should not be answered.

DOMAIN 1: Participants			
**A. Risk of Bias**			
Describe the sources of data and criteria for participant selection:<br>*Observational study with retrospective cohort based on SEER database including patients with urothelial bladder cancer who received radical cystectomy and lymph node dissection, during the years 2004-2019.*			
		Dev	Val
1.1 Were appropriate data sources used, e.g. cohort, RCT or nested case-control study data?		Y	Y
1.2 Were all inclusions and exclusions of participants appropriate?		Y	Y
**Risk of bias introduced by selection of participants**	**RISK:**<br>*(low/ high/ unclear)*	low	low
Rationale of bias rating:<br>*Cohort study with clear inclusion and exclusion criterial.*			
**B. Applicability**			
Describe included participants, setting and dates:<br>*Observational study with retrospective cohort based on SEER database including patients with urothelial bladder cancer who received radical cystectomy and lymph node dissection, during the years 2004-2019.*			
**Concern that the included participants and setting do not match the review question**	**CONCERN:**<br>*(low/ high/ unclear)*	low	low
Rationale of applicability rating:<br>*Included patients appear representative of the population specified in the review question*			

<table>
<tr><th colspan="4">DOMAIN 2: Predictors</th></tr>
<tr><th colspan="4">A. Risk of Bias</th></tr>
<tr><td colspan="4">List and describe predictors included in the final model, e.g. definition and timing of assessment:
The predictors included in the model were: AJCC stage, age, race, year of diagnosis, sex, T stage and summary stage.
All the predictors were measured at diagnosis.</td></tr>
<tr><td colspan="2"></td><td>Dev</td><td>Val</td></tr>
<tr><td colspan="2">2.1 Were predictors defined and assessed in a similar way for all participants?</td><td>Y</td><td>Y</td></tr>
<tr><td colspan="2">2.2 Were predictor assessments made without knowledge of outcome data?</td><td>PY</td><td>PY</td></tr>
<tr><td colspan="2">2.3 Are all predictors available at the time the model is intended to be used?</td><td>PY</td><td>PY</td></tr>
<tr><td>Risk of bias introduced by predictors or their assessment</td><td>RISK:
(low/ high/ unclear)</td><td>Low</td><td>Low</td></tr>
<tr><td colspan="4">Rationale of bias rating:
No risk of bias</td></tr>
<tr><th colspan="4">B. Applicability</th></tr>
<tr><td>Concern that the definition, assessment or timing of predictors in the model do not match the review question</td><td>CONCERN:
(low/ high/ unclear)</td><td>Low</td><td>Low</td></tr>
<tr><td colspan="4">Rationale of applicability rating:
No major issues identified</td></tr>
</table>

**DOMAIN 3: Outcome**			
**A. Risk of Bias**			
Describe the outcome, how it was defined and determined, and the time interval between predictor assessment and outcome determination:<br>*Cancer-specific survival was identified by SEER cause specific death*			
		Dev	Val
3.1 Was the outcome determined appropriately?		PY	PY
3.2 Was a pre-specified or standard outcome definition used?		Y	Y
3.3 Were predictors excluded from the outcome definition?		Y	Y
3.4 Was the outcome defined and determined in a similar way for all participants?		PY	PY
3.5 Was the outcome determined without knowledge of predictor information?		PY	PY
3.6 Was the time interval between predictor assessment and outcome determination appropriate?		Y	Y
**Risk of bias introduced by the outcome or its determination**	**RISK:**<br>*(low/ high/ unclear)*	Low	Low
Rationale of bias rating:<br>*No major issues identified*			
**B. Applicability**			
At what time point was the outcome determined:<br>3 years<br><br>*If a composite outcome was used, describe the relative frequency/distribution of each contributing outcome:*<br>*N/A*			
**Concern that the outcome, its definition, timing or determination do not match the review question**	**CONCERN:**<br>*(low/ high/ unclear)*	Low	Low
Rationale of applicability rating:<br>*The outcome of the primary study matches the outcome of interest of the review*			

**DOMAIN 4: Analysis**		
**Risk of Bias**		
Describe numbers of participants, number of candidate predictors, outcome events and events per candidate predictor:<br>A total of 11834 candidates (2004–2019) obtained from the Surveillance, Epidemiology, and End Results (SEER) database were randomly split into development cohort (n = 7889) and validation cohort (n = 3945)<br><br>In the table 1 we have the 35 predictors.<br><br>EPV= 4824/35= 168		
Describe how the model was developed (for example in regards to modelling technique (e.g. survival or logistic modelling), predictor selection, and risk group definition):<br><br>"We have considered conditional inference trees for survival analysis with censored data, which do not assume the need for proportional hazards and have the flexibility to model curves with different shapes for identified groups of subjects. Such trees estimate a regression relationship by recursive binary partitioning in a conditional inference structure, which ensures adequate tree growth without the need for further cross-validation. To perform the predictive model, we selected 2/3 of the sample (derivation cohort), we confirmed the model's validity by applying the parameters at 1/3 of the remaining sample (validation cohort)."		
Describe whether and how the model was validated, either internally (e.g. bootstrapping, cross validation, random split sample) or externally (e.g. temporal validation, geographical validation, different setting, different type of participants):<br><br>"We have used the pec package to compare the predictive performance of our proposal with the covariate-free survival Kaplan-Meier model and the Cox regression model, through the error defined as the time dependent expected Brier score. We consider for its calculation 500 samples of size 7889 that are randomly obtained from our database with 11834 records by means of a bootstrap cross-validation process"		
Describe the performance measures of the model, e.g. (re)calibration, discrimination, (re)classification, net benefit, and whether they were adjusted for optimism:<br><br>"We have used the pec package to compare the predictive performance of our proposal with the covariate-free survival Kaplan-Meier model and the Cox regression model, through the error defined as the time dependent expected Brier score. We consider for its calculation 500 samples of size 7889 that are randomly obtained from our database with 11834 records by means of a bootstrap cross-validation process"		
Describe any participants who were excluded from the analysis:<br>*No patients were excluded from the analysis.*		
Describe missing data on predictors and outcomes as well as methods used for missing data:<br>"We have used Multiple Imputation by Chained Equations (MICE) for the treatment of missing values"		
	Dev	Val
4.1 Were there a reasonable number of participants with the outcome?	Y	Y
4.2 Were continuous and categorical predictors handled appropriately?	Y	PY
4.3 Were all enrolled participants included in the analysis?	Y	Y
4.4 Were participants with missing data handled appropriately?	Y	Y

4.5 Was selection of predictors based on univariable analysis avoided?		Y	
4.6 Were complexities in the data (e.g. censoring, competing risks, sampling of controls) accounted for appropriately?		PY	PY
4.7 Were relevant model performance measures evaluated appropriately?		Y	Y
4.8 Were model overfitting and optimism in model performance accounted for?		Y	
4.9 Do predictors and their assigned weights in the final model correspond to the results from multivariable analysis?		PY	
**Risk of bias introduced by the analysis**	**RISK:** *(low/ high/ unclear)*	Low	Low
Rationale of bias rating: *No risk of bias.*			

Step 4: Overall assessment

Use the following tables to reach overall judgements about risk of bias and concerns regarding applicability of the prediction model evaluation (development and/or validation) across all assessed domains.
Complete for each evaluation of a distinct model.

Reaching an overall judgement about risk of bias of the prediction model evaluation	
**Low risk of bias**	If all domains were rated low risk of bias.<br>If a prediction model was developed without any external validation, and it was rated as low risk of bias for all domains, consider downgrading to **high risk of bias**. Such a model can only be considered as low risk of bias, if the development was based on a very large data set and included some form of internal validation.
**High risk of bias**	If at least one domain is judged to be at **high risk of bias**.
**Unclear risk of bias**	If an unclear risk of bias was noted in at least one domain and it was low risk for all other domains.

Reaching an overall judgement about applicability of the prediction model evaluation	
**Low concerns regarding applicability**	If low concerns regarding applicability for all domains, the prediction model evaluation is judged to have **low concerns regarding applicability**.
**High concerns regarding applicability**	If high concerns regarding applicability for at least one domain, the prediction model evaluation is judged to have **high concerns regarding applicability**.
**Unclear concerns regarding applicability**	If unclear concerns (but no "high concern") regarding applicability for at least one domain, the prediction model evaluation is judged to have **unclear concerns regarding applicability** overall.

Overall judgement about risk of bias and applicability of the prediction model evaluation		
**Overall judgement of risk of bias**	**RISK:**<br>*(low/ high/ unclear)*	Low
Summary of sources of potential bias:<br>No major issues identified		
**Overall judgement of applicability**	**CONCERN:**<br>*(low/ high/ unclear)*	Low
Summary of applicability concerns:<br>No major issues identified		
